# Development and Validation of a Statistical Shape Modeling-Based Finite Element Model of the Cervical Spine Under Low-Level Multiple Direction Loading Conditions

**DOI:** 10.3389/fbioe.2014.00058

**Published:** 2014-11-27

**Authors:** Todd L. Bredbenner, Travis D. Eliason, W. Loren Francis, John M. McFarland, Andrew C. Merkle, Daniel P. Nicolella

**Affiliations:** ^1^Musculoskeletal Biomechanics Section, Materials Engineering Department, Southwest Research Institute, San Antonio, TX, USA; ^2^Probabilistic Mechanics Section, Materials Engineering Department, Southwest Research Institute, San Antonio, TX, USA; ^3^Applied Physics Laboratory, The Johns Hopkins University, Laurel, MD, USA

**Keywords:** verification, validation, statistical shape modeling, finite element modeling, probabilistic analysis, cervical spine, injury

## Abstract

Cervical spinal injuries are a significant concern in all trauma injuries. Recent military conflicts have demonstrated the substantial risk of spinal injury for the modern warfighter. Finite element models used to investigate injury mechanisms often fail to examine the effects of variation in geometry or material properties on mechanical behavior. The goals of this study were to model geometric variation for a set of cervical spines, to extend this model to a parametric finite element model, and, as a first step, to validate the parametric model against experimental data for low-loading conditions. Individual finite element models were created using cervical spine (C3–T1) computed tomography data for five male cadavers. Statistical shape modeling (SSM) was used to generate a parametric finite element model incorporating variability of spine geometry, and soft-tissue material property variation was also included. The probabilistic loading response of the parametric model was determined under flexion-extension, axial rotation, and lateral bending and validated by comparison to experimental data. Based on qualitative and quantitative comparison of the experimental loading response and model simulations, we suggest that the model performs adequately under relatively low-level loading conditions in multiple loading directions. In conclusion, SSM methods coupled with finite element analyses within a probabilistic framework, along with the ability to statistically validate the overall model performance, provide innovative and important steps toward describing the differences in vertebral morphology, spinal curvature, and variation in material properties. We suggest that these methods, with additional investigation and validation under injurious loading conditions, will lead to understanding and mitigating the risks of injury in the spine and other musculoskeletal structures.

## Introduction

Cervical spine injuries are of significant concern in all trauma injuries, particularly given the potential for spinal cord injury in unstable injuries, with an estimated 42% of all cervical spine injuries being unstable (Milby et al., 2008). Recent military conflicts (i.e., Iraq and Afghanistan) have demonstrated the substantial risk of spinal injuries for the modern warfighter due to the high occurrence of traumatic injuries in combat. Explosive mechanisms resulted in inertial injuries due to direct blast injury and/or subsequent impacts in 75–78% of combat casualties (Belmont et al., 2012) and 28–39% of these injuries were suffered in the head/neck (Wade et al., 2007; Owens et al., 2008; Belmont et al., 2012). Although muscle strain is the most common injury in the neck, warfighters have experienced vertebral compression fractures and fracture of the spinous process in the lower cervical vertebrae, as well as interspinous ligament injuries in the lower cervical spine (Anderson, 1988; Schall, 1989; Coakwell et al., 2004).

Mechanisms of bony or ligamentous injury in the cervical spine and elsewhere have been investigated using finite element modeling, a common tool of structural analysts. Finite element models of the spine or spinal motion segments often employ generalized anatomical geometry or subject-specific geometry where the morphology of spinal components (e.g., vertebrae, intervertebral disks, and ligamentous structures) is described based on a specific set of imaging data, in either an explicit or idealized manner (Kallemeyn et al., 2010). However, such generic or individualized models do not allow for investigation of the full range of effects of variation in vertebral and spinal segment orientation and geometry and the influence of geometrical factors on the mechanical behavior of the spine (Laville et al., 2009).

Statistical shape modeling methods have been used to describe variability in the morphology of a population of anatomical structures in terms of a random field representation (Cootes et al., 1994; Lorenz and Krahnstover, 2000; Kaus et al., 2003; Rueckert et al., 2003). Current applications of statistical shape modeling (SSM) include automated image segmentation, image or object registration, object recognition, and disease diagnosis (Rueckert et al., 2003; Benameur et al., 2005; Dornaika and Ahlberg, 2006; Ferrarini et al., 2006; Rao et al., 2006; Koikkalainen et al., 2007). Statistical shape models capture the variability of biological structures by projecting a high dimensional representation of the structure onto a lower dimensional subspace of possible shapes constructed from a population of training shapes. A modeling approach combining SSM with finite element modeling allows investigation of loading behavior in specific individuals, as well as over the full range of morphological variability described within the model set.

The objectives of this study were to develop and implement methods based on SSM to describe the multivariate morphology and geometry within 3D imaging data for a set of cervical spines, to extend this model to a parametric finite element model of the cervical spine, and to quantitatively validate the performance of the parametric model against non-destructive experimental data. This study is the first step in this research program with the overall goal of investigating the effects of geometry and morphometry variation on the complex risks associated with cervical injury in scenarios that might be experienced in the occupational exposure of the modern warfighter, with the intent to provide the basis for mitigating these injuries in further work.

## Methods

### Image processing

**Figure 1:**
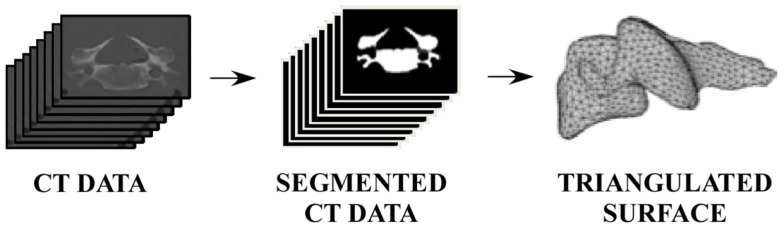
**Image processing pathway from CT data to segmented data to vertebral surface**.

Five cadaver specimens representative of the 50th percentile male warfighter (based on weight) were obtained and the cervical (C3–T1) spine of each specimen was scanned using a computed tomography (CT) system (Aquilion 64, Toshiba – Medical Systems, Tokyo, Japan). CT data were filtered using a sequence of median and anisotropic diffusion filtering to reduce data noise. Filtered data were semi-automatically segmented to extract vertebra data from the CT image data (Figure 1) (Seg3D, The Center for Integrative Biomedical Computing, University of Utah, Salt Lake City, UT, USA). Watertight triangulated surfaces were generated to describe the outer boundary of each vertebra (e.g., five cervical spines × seven vertebrae) by computing the isosurface geometry for the segmented data region and smoothing the resulting surfaces to remove any stair-stepping effects due to out of plane image resolution (Figure 1) (MATLAB R2012a, The Mathworks, Inc., Natick, MA, USA). Vertebral surfaces were resampled, resulting in approximately 4,000 faces for each triangulated surface.

### Development of individual spine models

**Figure 2:**
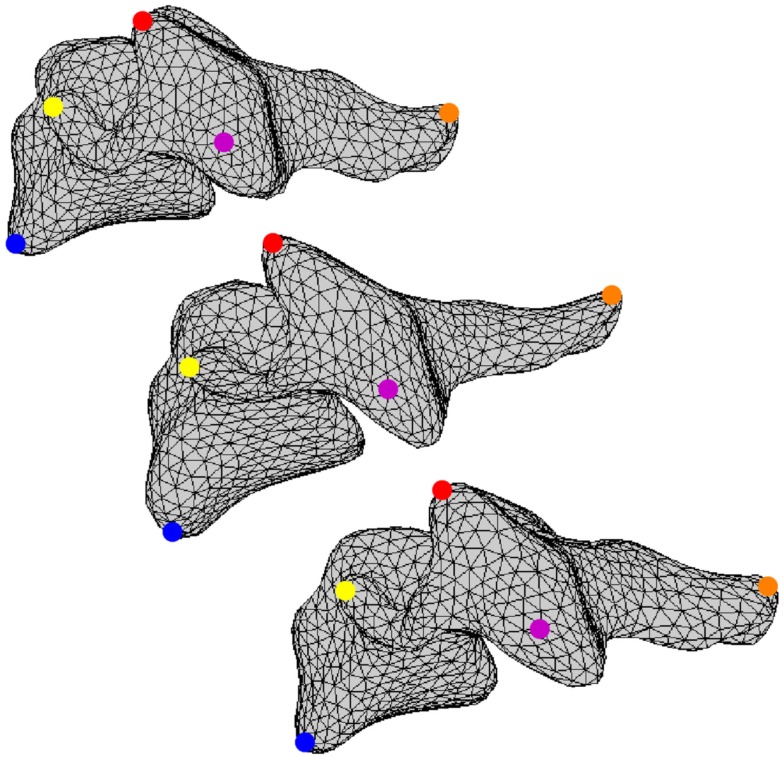
**A set of corresponding vertices is identified using blue, yellow, red, magenta, and orange dots on three different vertebral surfaces**.

All vertebral surfaces were positioned based on visual observation to yield nominal anatomic orientation of the full cervical spines and to correct for any positioning errors present during CT scanning (Scheer et al., 2013). The five surfaces at each vertebral level were registered to each other using an arbitrarily selected vertebral surface as the template. Vertices from the template surfaces were mapped onto the remaining four surfaces at each vertebral level and repositioned using a coherence point drift algorithm such that all vertices were positioned at corresponding anatomic locations between all vertebral surfaces at the same cervical level (Figure 2) (Myronenko and Song, 2010). Thus, the resulting vertebral surfaces were defined by the same surface mesh definition due to vertex correspondence across the set of vertebrae at each vertebral level. Average vertebrae were determined by averaging vertex positions for all vertebrae at each vertebral level.

Volumetric tetrahedral meshes consisting of 25,000–35,000 elements (5,500–6,800 nodes) were defined for the average vertebral surfaces and refined to improve mesh quality (Tetgen, Weierstrass Institute for Applied Analysis and Stochastics, Berlin, Germany; Stellar, University of California, Berkeley, CA, USA). At each vertebral level, the average vertebral mesh was elastically warped to match each individual vertebra using displacement vectors calculated between corresponding surface vertices on the average vertebral mesh and each individual vertebral surface, resulting in a set of five corresponding vertebral mesh models for each cervical level (ANSYS v11.0, ANSYS, Inc., Canonsburg, PA, USA). Individual corresponding vertebral meshes were transformed back to the nominal anatomic position of the appropriate vertebra within each cervical spine.

**Figure 3:**
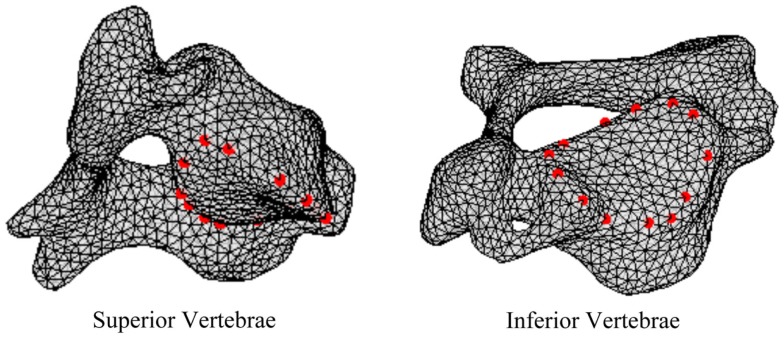
**Surface node sets were selected on adjacent vertebrae to specify the boundary of the intervertebral disk**.

**Figure 4:**
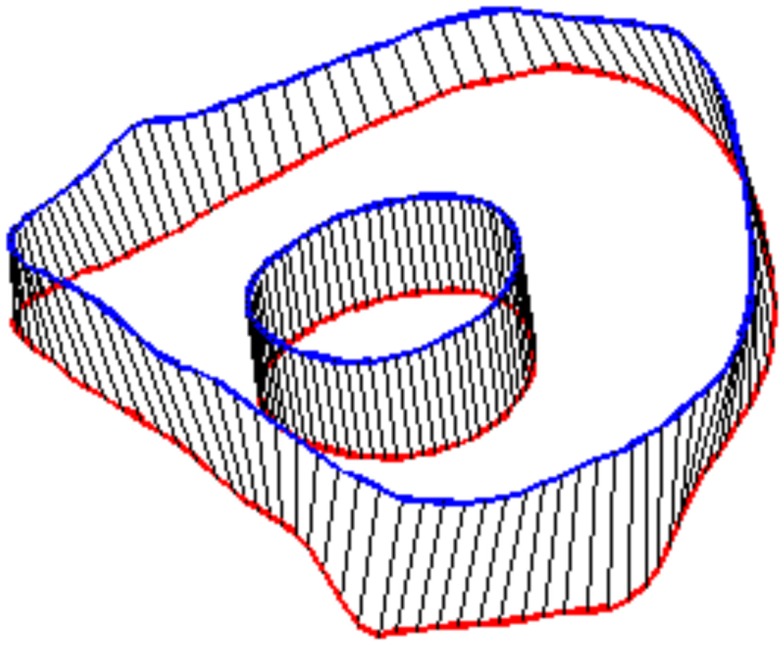
**Splines were fit to the sets of nodes defining intervertebral disk boundaries on adjacent vertebrae and the resulting splines were projected to the vertebral endplates**. Similarly, an ellipse was defined to represent the interface of the disk nucleus and annulus and projected to the adjacent vertebral endplates. Surfaces were defined by connecting the resulting closed curves and adjacent vertebral endplates further bounded the intervertebral disk space.

**Figure 5:**
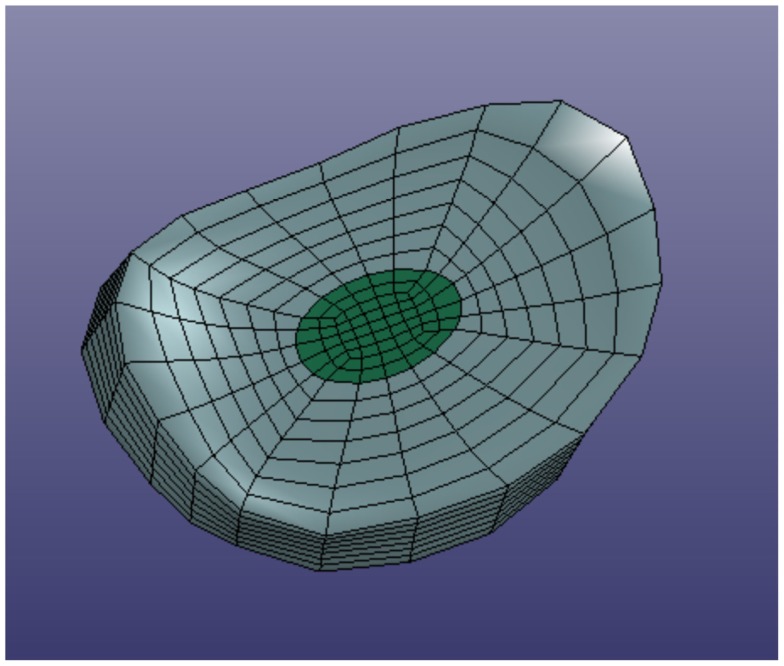
**Typical intervertebral disk model with separate annulus and nucleus models**.

In order to define intervertebral disk models, vertices bounding the approximate location of the intervertebral disk were selected on the adjacent vertebral endplates of the average cervical spine model (Figure 3). Due to mesh correspondence, vertices selected on the average motion segment models defined disk boundaries for all individual motion segments. Splines were fit through each set of selected surface vertices, resulting in a set of closed curves lying on the respective endplate surface and defining disk boundaries on adjacent endplates of the individual cervical spine models (Figure 4). Ellipses with dimensions proportional to the disk boundary curves were defined at the centroid of adjacent endplate curves and projected on to adjacent surfaces in order to define the interface between disk nucleus and annulus (Figure 4). Surfaces defining outer boundaries of the disk annulus and nucleus were generated between appropriate splines on adjacent vertebral endplates and disk endplate surfaces further bounded the intervertebral disk space. A hexahedral mesh of the intervertebral disks was defined within the bounded disk space for each individual spines (Truegrid, XYZ Scientific Applications, Inc., Livermore, CA, USA) (Figure 5).

Facet joint cartilage was modeled by projecting the set of surface triangles that define facet surfaces of adjacent vertebrae outward along the vertex normals to form a single layer of wedge elements. Facet cartilage elements were defined for each facet surface using a constant thickness, which was iteratively determined to maximize joint contact without facet surface interference. Ligaments (e.g., anterior longitudinal ligaments, posterior longitudinal ligaments, interspinous ligaments, ligamentum flavum, and intertransverse ligaments) and facet joint capsules were modeled using discrete spring elements to connect selected nodes on adjacent vertebrae. The facet regions and nodes representing ligament attachment sites were determined from the average cervical spine mesh. Mesh correspondence allowed the same sets of surface triangles and nodes to be used on each of the individual spine models.

**Figure 6:**
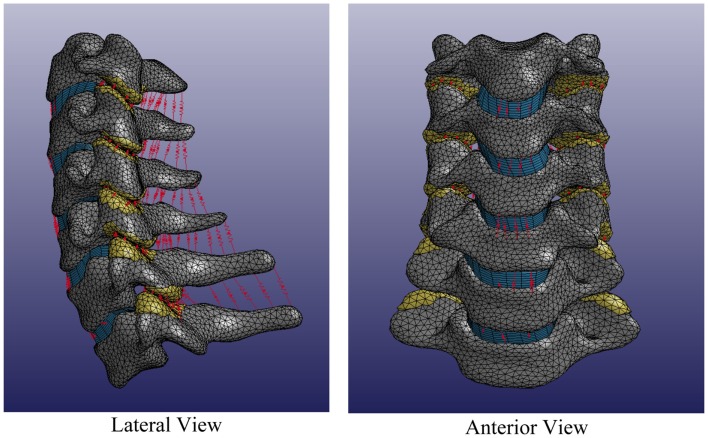
**Average cervical spine model note: vertebral bodies are shown in gray, intervertebral disks are shown in blue, facet elements are shown in yellow, and ligaments are shown in red**.

Individual spines were translated such that the centroid of the central vertebra (e.g., C4) was located at the origin of the Cartesian space in order to register individual spines while maintaining individual spine intersegmental spacing and curvature. Each individual volumetric model of the spine included C3–T1 vertebrae, intervertebral disks, facet joints, and relevant ligamentous structures (Figure 6).

### Development of statistical shape models of the cervical spine

A SSM was generated to describe and investigate geometric variability in the set of five cervical spines. Joint point distribution models were constructed from all individualmeshes. The volumetric mesh for each individual was described by a shape parameter vector as:

(1) $${\text{p}}_{\text{i}}={\left(v_{1x},v_{1y},v_{1z},\ldots ,v_{jx},v_{jy},v_{jz}\right)}^{T}$$

where *v_j_*_(_*_xyz_*_)_ are the three-dimensional coordinates of the nodes in the volumetric spine model, *j* = 1, … , *J* = 54,960 nodes in the volumetric mesh, and *i* = 1, … , *n* = *5* denote each individual spine in the set.

The mean shape of all components (e.g., vertebrae, disks, facets, and ligament attachment points) in the set of cervical vertebrae was defined as:

(2) $$\bar{\text{p}}=\frac{1}{n}\sum_{i=1}^{n}{\text{p}}_{i}$$

and the correlation between individual models in the set was given by the empirical covariance matrix:

(3) $$\text{S}=\frac{1}{n}\sum_{i=1}^{n}\left({\text{p}}_{i}-\bar{\text{p}}\right){\left({\text{p}}_{i}-\bar{\text{p}}\right)}^{T}$$

A principal components analysis (PCA) of the covariance matrix, ***S***, results in a set of *k* = *n* − 1 eigenvalues (λ*_k_*) and eigenvectors (***q****_k_*), which are the principal directions spanning a shape space centered at the mean, $\bar{\text{p}}$. The proportion of the total variance described along each eigenvector is equal to its corresponding eigenvalue divided by the sum of all eigenvalues; eigenvectors corresponding to the largest eigenvalues describe the majority of the variance. Thus, the finite element mesh for each cervical spine in the set were described in terms of the average model and a weighted linear combination of uncorrelated principal shape modes as:

(4) $${\text{p}}_{v}=\bar{\text{p}}+\sum_{j=1}^{m}c_{j}\sqrt{\lambda_{j}}{\text{q}}_{j}$$

where ***p_v_*** is a vector containing coordinates for all nodes in the FE model, *m* is the number of eigenvalues, *λ_j_*, and deviation from the average spine, $\bar{\text{p}}$, was determined as the sum of the products of a set of scalar weighting factors, ***c_j_***, and SSM standard deviations, $\sqrt{\lambda_{j}},$ along the ***q_j_*** (eigenvector) directions (Bredbenner et al., 2010; Nicolella and Bredbenner, 2012).

Accordingly, the highly correlated 3D spine geometry variables are reduced into a relatively small set of uncorrelated and independent composite morphological traits. All variability within the original set of spine models (originally described by over 164,880 variables) is now described by the weighting factors for four principal components for each cervical spine. Principal components are new descriptive variables that, by definition, are linear combinations of the original descriptive variables and, furthermore, all geometry information in the original models is retained in the new model descriptions.

In order to investigate the variability in vertebral morphology, intersegmental orientation, and overall curvature in the cervical spine models, a series of variation models were created and compared to the average models. Principal component weighting factors in Eq. 4 were modified to generate models describing the difference of 1.0 standard deviation of each principal component (i.e., shape mode) from the average model.

### Development of a parametric finite element model of the cervical spine

The statistical shape model of the cervical spine (including vertebrae, disks, and ligaments) was generated in a form directly applicable to finite element analysis and geometry variation in the finite element model was explicitly described by principal component weighting factors. The average spine segment model was created. Additionally, the effects of spine geometry variation were investigated by modifying the average model with the geometry traits carried by the principal components (Eq. 4). Principal component weighting factors were defined as random variables with a mean, standard deviation, and distribution shape (Table 1). Vertebrae were modeled as rigid bodies, as this investigation did not consider vertebral fractures.

**Table 1:** **Random variable definitions**.

Random variables	Value	Distribution type
Weighting factor for PC1	0.0 ± 1.0 (−0.9–0.9)	Truncated normal
Weighting factor for PC2	0.0 ± 1.0 (−0.9–0.9)	Truncated normal
Weighting factor for PC3	0.0 ± 1.0 (−0.9–0.9)	Truncated normal
Weighting factor for PC4	0.0 ± 1.0 (−0.9–0.9)	Truncated normal
Bulk modulus for intervertebral disks	10.99 ± 8.47 MPa	Lognormal
Scale factor for ALL load curve (C3–C5)	0.2500 ± 0.0422	Lognormal
Scale factor for ALL load curve (C5–T1)	0.2500 ± 0.0480	Lognormal
Scale factor for PLL load curve (C3–C5)	0.2500 ± 0.0709	Lognormal
Scale factor for PLL load curve (C5–T1)	0.2500 ± 0.0260	Lognormal
Scale factor for ISL load curve (C3–C5)	0.2500 ± 0.0520	Lognormal
Scale factor for ISL load curve (C5–T1)	0.2000 ± 0.0211	Lognormal
Scale factor for LF load curve (C3–C5)	0.1250 ± 0.0352	Lognormal
Scale factor for LF load curve (C5–T1)	0.1250 ± 0.0211	Lognormal
Scale factor for JC load curve (C3–C5)	0.1250 ± 0.0206	Lognormal
Scale factor for JC load curve (C5–T1)	0.1250 ± 0.0205	Lognormal

*PC, principal component; ALL, anterior longitudinal ligament; PLL, posterior longitudinal ligament; ISL, interspinous ligament; LF, ligamentum flavum; JC, joint capsule*.

**Figure 7:**
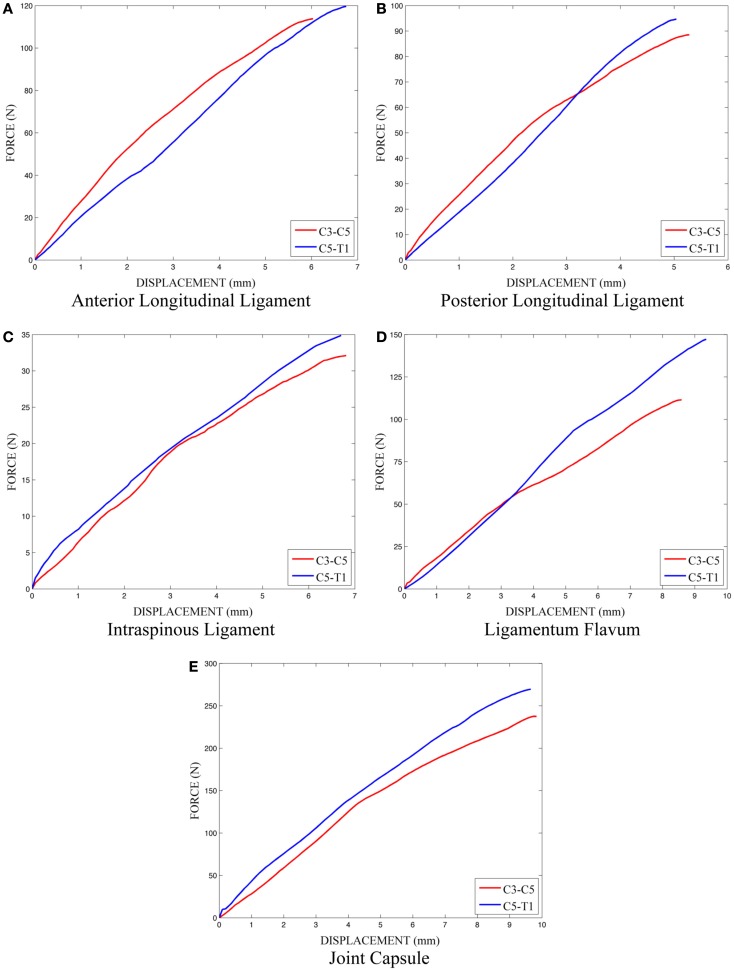
**Experimental load-displacement data for soft-tissue material behavior**.

The effects of variation and uncertainty in intervertebral disk and soft-tissue material properties were also investigated by considering appropriate material parameters as random variables. Each disk was modeled with a separate annulus and nucleus. Soft-tissue material properties were modeled based on experimental data found in the literature. Material behavior of the annulus was modeled with a transversely isotropic hyperelastic model with viscosity and material parameters were determined from experimental data provided by Lucas et al. (2006). Lucas et al. collected experimental data for the annulus using a ramp and hold loading protocol, with a high rate ramp (52 mm/s) to 0.88 mm and a 10-s hold period, and also under sinusoidal loading conditions (2 Hz with 0.65 mm peak-to-peak displacement). Material properties used in the present model were determined using the relaxation data and validated successfully against the dynamic loading data. The bulk modulus of the annulus was defined as a random variable (Table 1). A Prony series was defined to approximate the viscoelastic relaxation behavior of the annulus (Table 2). Material behavior of the nucleus was modeled using a fluid material model and material properties were determined from the literature (Table 3) (Teo and Ng, 2001; Nicolella et al., 2006). Material behavior for the ligamentous and capsular structures was defined using experimental force-displacement data that were collected under quasi-static loading conditions (Yoganandan et al., 2000) (Figure 7). As multiple spring elements were used to model anterior longitudinal ligaments, posterior longitudinal ligaments, interspinous ligaments, ligamentum flava, and joint capsules, experimental soft-tissue force-displacement data were scaled to account for distribution of the soft-tissue response over multiple discrete elements. Soft-tissue scale factors were treated as random variables (Table 1).

**Table 2:** **Viscoelastic properties for the intervertebral disk annulus**.

*i*:	1	2	3	4	5
**C3–C5**
*S_i_*	0.7440	0.1098	0.0356	0.0251	0.0855
*τ_i_*	0.001	0.01	0.1	1.0	1000.0
**C5–T1**
*S_i_*	0.7440	0.1098	0.1580	0.0251	0.1053
*τ_i_*	0.001	0.01	0.1	1.0	1000.0

**S_i_*, Prony series coefficients defining the relaxation function; *τ_i_*, characteristic time constants for the prony series, where values are given in seconds. Viscoelastic properties were obtained from Lucas et al. (2006)*.

**Table 3:** **Material properties for the intervertebral disk nucleus**.

Bulk modulus (MPa)	Poisson’s ratio	Viscosity coefficient
2.19	0.49	0.3

*Material properties were obtained from Teo and Ng (2001)*.

The effects of variation in spine geometry and soft-tissue material properties on cervical spine kinematic response were determined by sampling the appropriate variable distributions 100 times using a Latin Hypercube approach within a probabilistic framework (NESSUS v8.0, Southwest Research Institute, San Antonio, TX, USA). Additionally, the contributions of each principal component to model loading response were investigated by creating the average model and models created by modifying the average model with the geometry traits described by +1 standard deviation of each principal component (Eq. 4), where principal components were considered individually. Disk and soft-tissue material properties were defined using mean values. In all model simulations, T1 was fixed and pure flexion-extension, left–right axial rotation, or left–right lateral bending moments of ±2.0 Nm were applied to C3 and models were solved using LS-DYNA v. 971 (LSTC, Livermore, CA, USA).

### Validation of the parametric finite element model of the cervical spine

A hierarchical probabilistic verification and validation approach was used to quantify the performance of the parametric cervical spine model (ASME, 2006; Nicolella et al., 2006). Briefly, in previous work, material model parameters for the various ligaments and intervertebral disk components were determined and validated and the kinematic response of each motion segment (e.g., C3–C4, C4–C5, and C6–C7) was validated against independent experimental data without any alteration to the material model parameters. In the present work, the performance of the full C3–T1 parametric spine model was validated against independent experimental response data collected using cervical (C2–T1) specimens obtained from seven “normal” young human cadaver specimens (five males and two females; aged 33.4 ± 11.7 years with a range of 20–51 years) provided by Wheeldon et al. (2006). Ligamentous soft tissue was left intact in the experimental specimens. Wheeldon et al. fixed the T1 vertebra to a six-axis load cell, preconditioned the spine segment, and applied quasi-static pure moments of 0.33, 0.5, 1.0, 1.5, and 2.0 Nm to C2, resulting in cervical spine flexion-extension. Reaction load and vertebral rotation data were recorded throughout the experimental testing. Wheeldon et al. (unpublished data) used an identical loading protocol to load the cervical segments in left–right axial rotation and left–right lateral bending.

In order to evaluate the predictive performance of the parametric cervical spine model under non-destructive loading conditions, the mean and one-standard deviation load response envelopes were qualitatively compared between the model predictions and the experimental data for each loading model. In order to quantify model performance, we implemented a general performance metric that characterizes the disagreement between the model variation and relevant experimental data variation (Ferson et al., 2008; Francis et al., 2012). This quantitative metric provides a generalized approach to validation, rather than focusing on comparison of the mean predicted and experimental behaviors (Ferson et al., 2008). Empirical cumulative distribution functions (CDFs) were fit to the experimental data obtained from seven cervical spine specimens at each of five applied moment values (0.33, 0.5, 1.0, 1.5, and 2.0 Nm) over the loading range for each loading mode (e.g., flexion-extension, axial rotation, and lateral bending). Empirical CDFs were also fit to the predicted loading response for each loading mode at identical applied moment values, where response data were determined for each of the models generated by 100 Latin Hypercube samples of the variable space. Area metric values were determined as the total area difference between the CDFs for the experimental results and the model predictions at each of the five applied moment values for each loading mode and have units of degrees, as with the loading response. However, metric values are directly associated with the loading level; therefore, metric values were normalized by the mean experimental rotational displacement at each loading point. The resulting dimensionless values were used to measure the agreement between the response distributions over the full range of loading for each loading mode.

## Results

The first principal component explains 63.8% of the total variability in cervical spine geometry, the first and second together explains 78.1%, and the first three principal components explain 90.3% of the geometric variability in the five cervical spine segments, with the remainder of the variability described by the fourth principal component.

**Figure 8:**
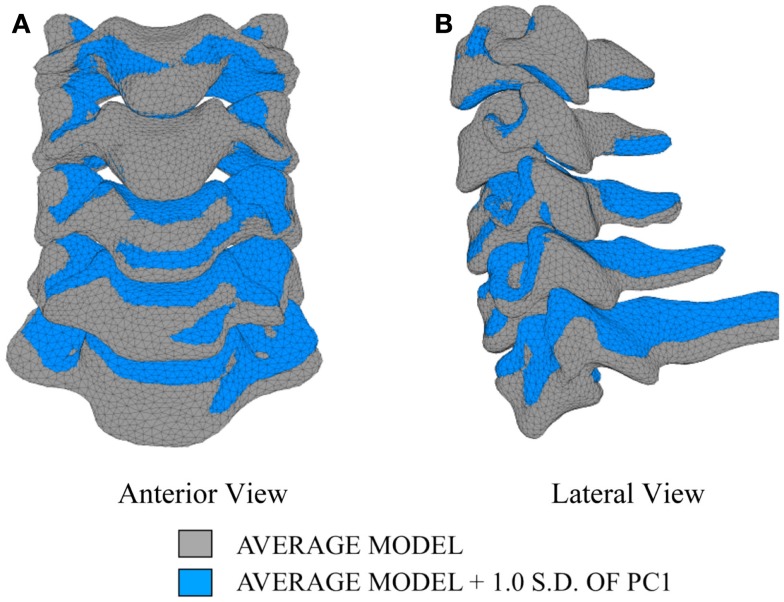
**Variation in geometry and alignment described by principal component (PC) 1**.

**Figure 9:**
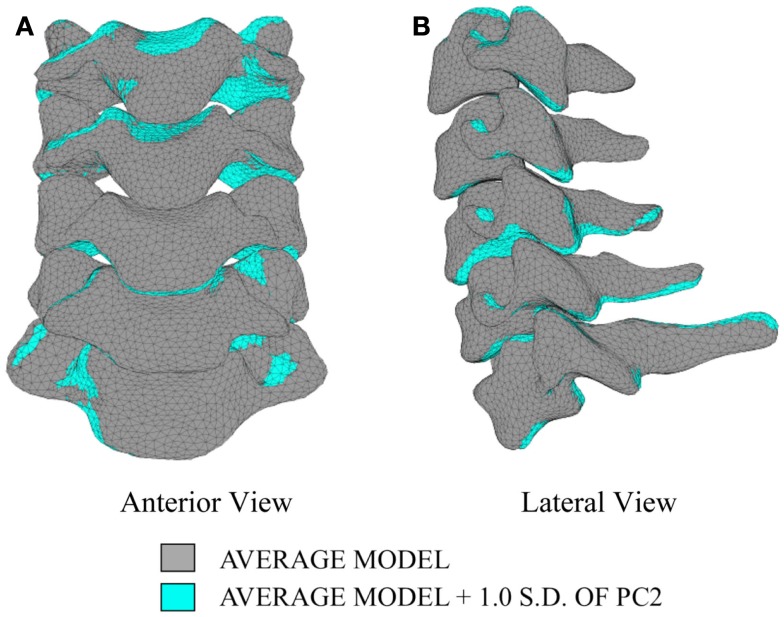
**Variation in geometry and alignment described by principal component (PC) 2**.

**Figure 10:**
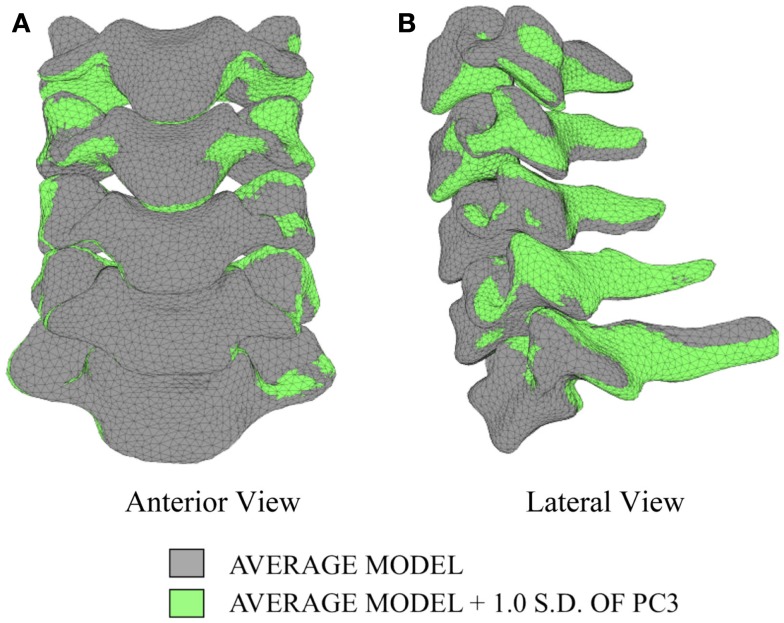
**Variation in geometry and alignment described by principal component (PC) 3**.

**Figure 11:**
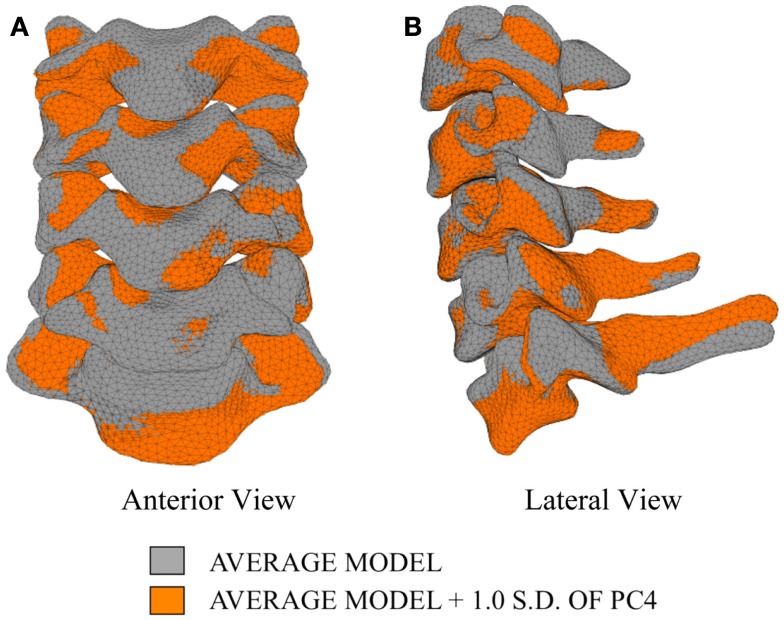
**Variation in geometry and alignment described by principal component (PC) 4**.

Differences in morphology of individual vertebrae, intersegmental orientation, and overall spinal curvature are evident in qualitative comparisons between the average and variation models of the cervical spine (Figures 8–11). Variations in intersegmental orientation in each of the three major axes are clearly visible and, in some cases, obscure variations in specific vertebral morphology, although differences in posterior process shape and length is most evident. The cumulative effects of intersegmental variation over the cervical spine lead to overall variation of the full cervical spine segment.

**Figure 12:**
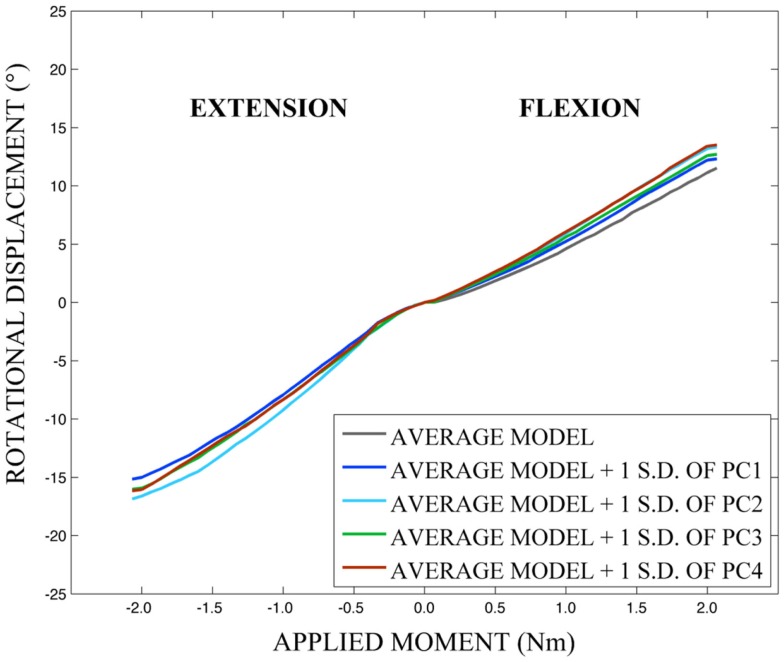
**Effects of geometry variation on flexion-extension**.

**Figure 13:**
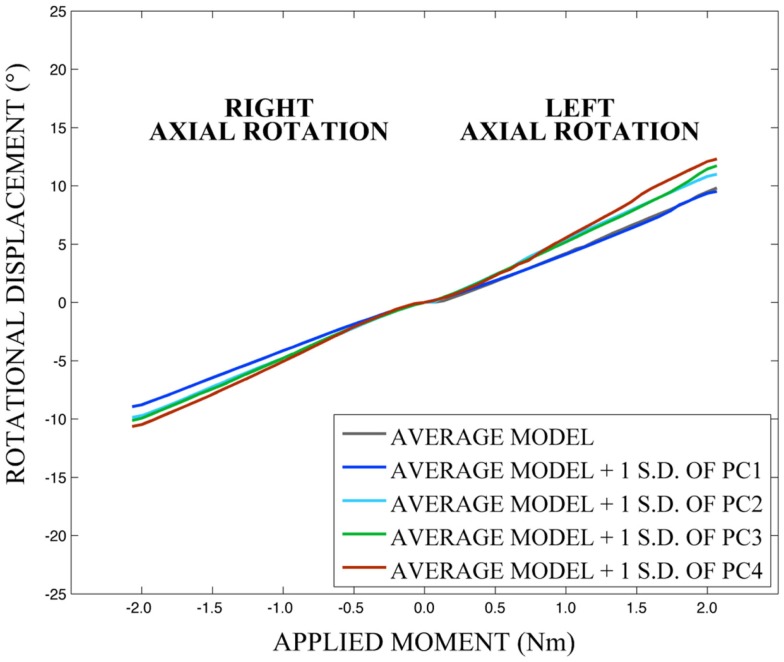
**Effects of geometry variation on axial rotation**.

**Figure 14:**
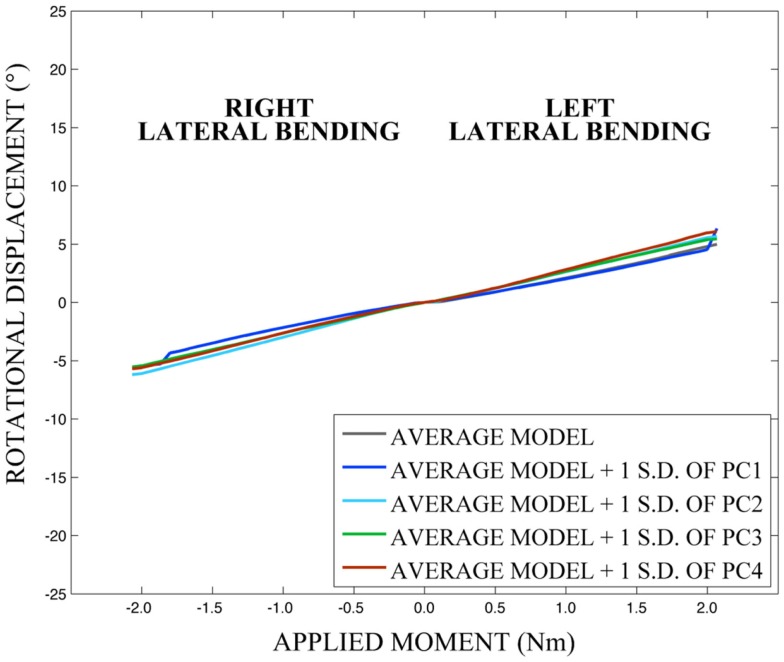
**Effects of geometry variation on lateral bending**.

As expected, investigation of the effects of spinal morphology variation on predicted loading response resulted in quantifiable variation in the loading responses between the average model and models created by combining the average model and the geometry variation described by +1.0 standard deviation of each principal component (Figures 12–14). Response variations were determined as percentage change with respect to the mean model. In the case of flexion-extension (Figure 12), principal component 4 had the largest effect on the flexion response (17.3%) and principal component 3 had the largest effect on extension (23.2%). Principal component 4 had the largest effect on axial rotation (Figure 13), with 19.5 and 25.5% variation for right and left rotation, respectively. In the case of lateral bending (Figure 14), principal component 3 had the largest effect on right bending (22.4%) and principal component 2 had the largest effect on left bending (27.1%).

**Figure 15:**
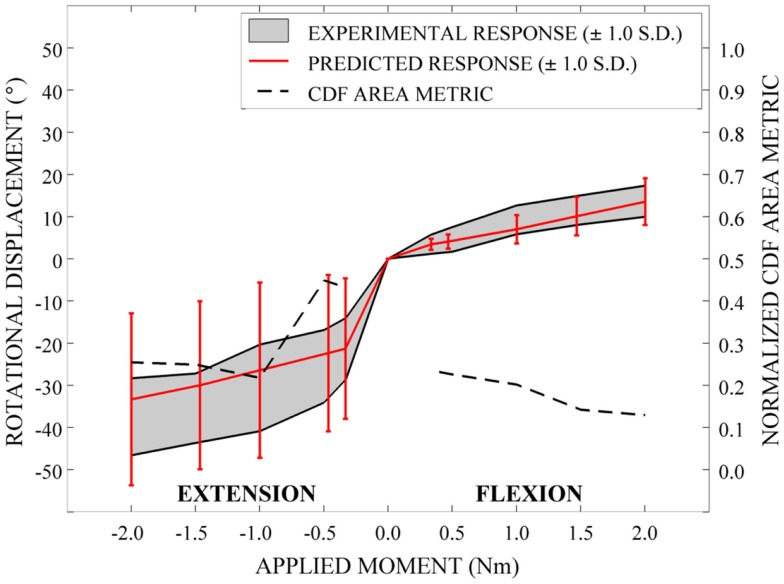
**Kinematic response and normalized CDF area metric for flexion-extension**.

**Figure 16:**
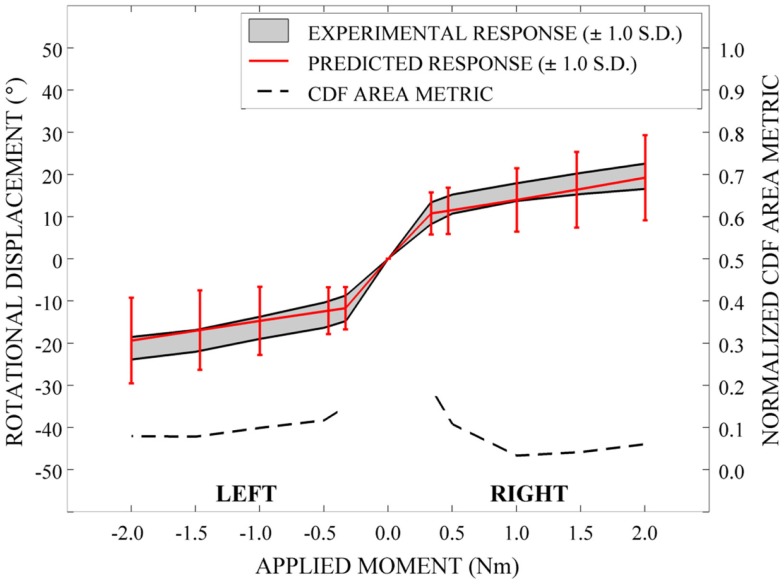
**Kinematic response and normalized CDF area metric for left-right axial rotation**.

**Figure 17:**
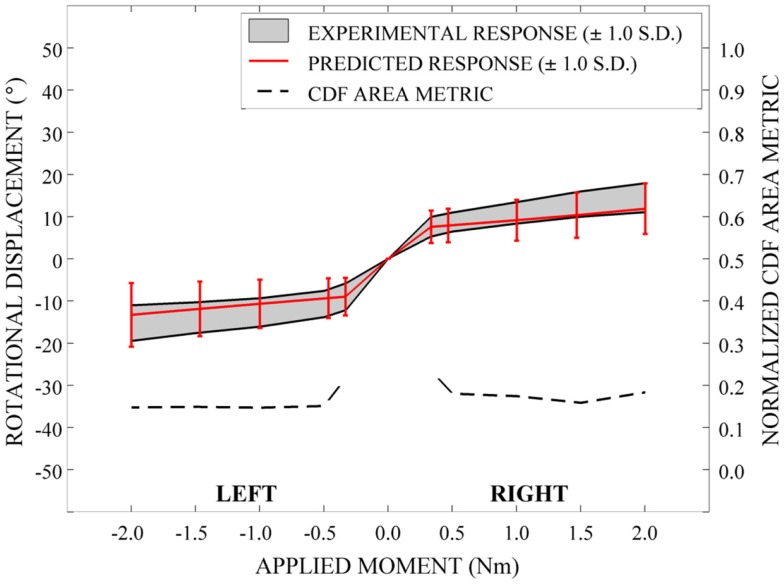
**Kinematic response and normalized CDF area metric for left-right lateral bending**.

Qualitatively, the mean and variation in the probabilistic results closely match those of the experimental results. In general (Figures 15–17), the variability of the predicted response is greater than that of the experimental response; however, the mean predicted response lies within the +1.0 standard deviation envelope for experimental data in all cases. The parametric model also predicts greater variation than the experimental results as the applied moment increases for each loading mode.

Disagreement between model and experimental CDFs were quantified using the normalized CDF area metric as between 0.22 and 0.45 for each time point in extension (Figure 15), between 0.03 and 0.19 for right axial rotation (Figure 16), and ranges between 0.13 and 0.24 for all time points for all other loading modes (Figures 15–17).

## Discussion

This study demonstrated that significant variability was present in the geometry of a small group of cervical spine segments, both in terms of vertebral morphometry and in the intervertebral orientation and overall spinal curvature. Furthermore, SSM is capable of efficiently describing variability in the complex vertebral morphometry, intersegmental orientation, and overall spinal curvature and demonstrates the complex relationship between predicted response and variation in model input parameters. Although we have investigated conditions within the non-destructive loading range of the cervical spine, we suggest that, with additional validation under destructive loading conditions, the present implementation of parametric SSM-based finite element analysis methods is applicable to investigations of cervical spine injury mechanisms. These loading scenarios include those that might be encountered during high-speed impact in a moving vehicle, as the result of an explosive blast, or the ejection of a fighter pilot. More importantly, the parametric high-fidelity description of variability in spinal morphology, along with the ability to vary relevant material properties within a probabilistic framework allows the investigation of the effects of body size, position, mass distribution, and other relevant factors on injury risk prediction.

Intersegmental orientation and spinal curvature is highly variable between individuals and has a substantial role in individual disposition to neck and back injuries, including muscular fatigue or soft-tissue injuries, as well as more serious injuries related to hyperflexion and hyperextension (Coakwell et al., 2004; Frechede et al., 2006). Variation in intersegmental orientation and overall spinal curvature are modeled implicitly in the current SSM approach; however, explicitly modeling vertebral orientation and spinal curvature variables would allow the investigation of the role and interaction of vertebral morphology, intersegmental orientation, and spinal curvature variables on the likelihood of injury under various loading conditions.

The small sample size of spines is a limitation of the present study and it remains to be seen whether the range of morphological differences observed within the study sample are sufficient to describe a larger population of warfighters. In this study, we choose to focus on the development of a model of the 50th percentile male as a starting point, with the intent to develop methodology somewhat representative of the loading response of an “average” male warfighter. The geometric and material property variation described in the cervical spine model may well be different than that within the experimental set of spines; however, probabilistic methods allowed investigation of the effects of variability in morphology and material properties on kinematic response. Based on qualitative and quantitative comparison of the model simulations to experimental loading response data that is currently available, we suggest that the model performs adequately under relatively low-level loading conditions in multiple loading directions. In ongoing work, the cervical spine model is being exercised and validated against destructive loading modeling blast conditions for the same set of spines used to construct the model. It remains to be determined whether differences in vertebral morphology and spine geometry and the ability to describe these differences using a small set of principal shape modes will be capable of future risk classification under the widely varying scenarios that may be encountered by a warfighter. We also note ligament material properties were defined based on quasi-static loading conditions and this may present a limitation in evaluation of the model under destructive, high rate loading conditions.

In earlier work involving a parametric cervical spine model based on idealized vertebral geometry, we have demonstrated that both morphological differences between small female, large female, small male, and large male groups (determined by body mass) and material properties of ligaments and the intervertebral disks have a substantial effect on the predicted kinematic response under loading modes (e.g., flexion-extension, axial rotation, and lateral bending) with identical applied moments (Nicolella et al., 2006). Other groups using idealized parametric models of models with subject-specific geometry have also found that geometry and orientation and material models employed in describing soft-tissue behavior in both cervical spine motion segments lead to differences in the loading response (Kumaresan et al., 1999; del Palomar et al., 2008; Laville et al., 2009; Kallemeyn et al., 2010). In other work, finite element models of the cervical spine were deformed to model gross global changes in curvature (e.g., lordosis, straight, and kyphosis models) based on specified Cobb angle (Frechede et al., 2006). Distributions of strains, forces, and moments along the cervical spine were found to be dependent on loading condition (e.g., vehicular rear-end, frontal, lateral, and oblique impact) and global cervical curvature affected the magnitude of strains, forces, and moments experienced by the spine in all loading directions. However, we are unaware of probabilistic investigations where SSM methods were combined with finite element analyses to systematically investigate the effects of the continuous normal geometric variation in a sample of spines and material property uncertainty on loading response in the cervical spine. We suggest that the ability to continuously vary vertebral morphology, intersegmental orientation, and spinal curvature along with soft-tissue material properties within realistic parameter spaces strongly enhances the current investigative tools employed to understand and mitigate the risk of fracture and soft-tissue injuries in military combat scenarios.

We note that in validation of the present model under low-loading conditions in multiple directions, uncertainty and variability in both experimental results and model inputs (and, therefore, model results) are considered stochastically through the determination of a empirical distributions to describe experimental results and the use of probabilistic modeling methods to determine computation results. Therefore, we have quantitatively compared the statistical distributions resulting from both simulation predictions and experimental observations (Liu et al., 2011). The CDF area metric employed here to quantify disagreement between model predictions and experimental data evaluates the ability of the model to not only predict the mean, but the amount of variation in the experimental response. Additionally, the metric prevents incorrect “validation” of model predictions based on experimental data with large corridors of uncertainty. At each time point for each loading mode, the CDF area metric is a quantitative measure of agreement between model predictions and experimental data with reliance on expert opinion to evaluate the accuracy of the predictions. This is relatively straightforward to understand since the metric can be displayed graphically and has an intuitive meaning. Furthermore, unlike other computational model validation approaches, the area metric incorporates variability in both the experiment and model. It has a minimum value of 0, indicating the experimental and model CDF are the same, and has an infinite maximum value with increasing values indicating an increasingly poor match. We note that the quantitative error metric provides relative error (rather than absolute error) within the range of data analyzed and is, of course, dependent on whether the experimental data used for comparison is representative of the conditions modeled. As such, the area-based metric is limited in the case where the experimental data are insufficient (Liu et al., 2011). We have not exercised the model to produce injury in this study, and therefore, have not fully explored the kinematic and dynamic range of the model and Wheldon et al. have not thoroughly exercised the experimental specimens (Wheeldon et al., 2006; unpublished data). Accordingly, we suggest that some concern regarding insufficiency of experimental data may be reduced.

A potential use of a quantitative metric, such as the one used in the current analysis, is to investigate the effect of the use of alternative sources of data for material constitutive modeling, the incorporation of additional validation data when available, and the comparison of alternative or competing models and modeling approaches. A limitation of the area validation metric, however, is that an established standard for what constitutes an “acceptable” validation threshold has not been established. Therefore, this and other validation metrics should be used within the context of the overall objectives and goals of the use of the computational model. Qualitative comparison of the mean and standard deviation envelopes of the experimental and simulated loading responses suggest that the amount of error realized in the simulations are acceptable for the applied loading conditions. The quantitative metric values in each loading mode suggest that error is greater near the so-called “slack region” close to an applied moment of 0 Nm, where the model is not explicitly accounting for the ligamentous laxity that exists within cadaveric specimens. We note that in subsequent investigations utilizing this parametric cervical spine model with injurious loading conditions, we will be less concerned with areas of laxity during low-level loading and more concerned with the peak regions of the loading range; however, it remains to be seen whether a similar pattern of error will be present over a larger loading range.

In conclusion, SSM provides a means of explicitly describing complete spinal morphology and geometry and allows the complex spatial variation in intersegmental orientation to be statistically investigated between individuals. SSM methods coupled with finite element analyses within a probabilistic framework, along with the ability to quantitatively validate the overall model performance, provides innovative and important steps toward describing differences in vertebral morphology, intersegmental orientation, and spinal position and curvature, as well as important variation in material properties, which may directly lead to understanding and mitigating the risks of soft-tissue and hard tissue injury in the spine and other musculoskeletal structures.

## Conflict of Interest Statement

The authors declare that the research was conducted in the absence of any commercial or financial relationships that could be construed as a potential conflict of interest.
